# Quality characteristics and flavor compounds of pork meat as a function of carcass quality grade

**DOI:** 10.5713/ajas.18.0965

**Published:** 2019-03-07

**Authors:** Van Ba Hoa, Pil-Nam Seong, Soo-Hyun Cho, Sun-Moon Kang, Yun-Seok Kim, Sung-Sil Moon, Yong-Min Choi, Jin-Hyoung Kim, Kuk-Hwang Seol

**Affiliations:** 1 National Institute of Animal Science, Rural Development Administration, Wanju 55365, Korea; 2 Sunjin Meat Research Center, Ansung 17532, Korea; 3 National Institute of Agricultural Sciences, RDA, Wanju 55365, Korea

**Keywords:** Quality Grade, Meat Quality, Fatty Acid, Flavor Compound, Sensory Quality

## Abstract

**Objective:**

The present work aimed at evaluating the effects of carcass quality grade (QG) on the quality characteristics of pork meat according to Korean carcass QG system.

**Methods:**

Pork carcasses with varying in QG: 1+ (QG1+, n = 10), 1 (QG1, n = 10) and 2 (QG2, n = 10), were used to evaluate the relationship between carcass QG and meat quality. The meat quality traits, fatty acid profiles, flavor compounds and sensory qualities were measured on the *longissimus dorsi* muscle samples of these carcasses.

**Results:**

Pork meat of higher QG (QG1+) presented significantly higher fat content (5.43%), C18:2n-6 level (19.03%) and total unsaturated fatty acids content (62.72%). Also, the QG1+ meat was significantly higher in levels of classes of flavor compounds such as aldehydes, alcohols and hydrocarbons in comparison to those of the meat samples from the lower QG groups. The sensory evaluation results (flavor, juiciness, tenderness, and acceptability scores) of QG1+ meat was significantly higher than the QG1 and QG2 meats. The pork with lower QG (i.e., QG2) was found positively correlated to redness (r = 0.987), C18:1n-9 level (r = 1.000) but negatively correlated to the fat content (r = −0.949), and flavor (r = −0.870), juiciness (r = −0.861), tenderness (r = −0.862) and acceptability (r = −0.815) scores.

**Conclusion:**

The pork with higher QG had higher fat content, total unsaturated fatty acids and better eating quality, thus producing pork with higher QGs should be considered in order to satisfy the consumer’s expectation.

## INTRODUCTION

Pork is one of the most consumed red meat types in many countries worldwide. In the past decades, the pork industry has strongly focused on genetic improvements for lean meat production [1]. Recently, the pork industry, however, is faced with a problem due to limited amount of visible fat in meat tissues because the production of leaner and heavier pigs has adverse effect on amount of intermuscular fat (IMF), which makes the pork tougher, less moist and with reduced flavor [2–4]. In some meat markets, the level of visible fat is the strongest visual discriminative factor entering in the purchasing decision process [5–7]. Studies on consumer’s perception and eating preferences for pork have indicated that a higher level of IMF or marbling is associated with better eating quality [6–10]. A recent study by Moeller et al [11] showed that consumers rated higher tenderness, juiciness and flavor scores for the high IMF pork compared to the low IMF pork. In contrast, a study by Rincker et al [12] reported that the level of IMF did not correlate strongly with eating quality such as tenderness, juiciness or pork flavor for pork loins. Therefore, further scientific evidences should be provided in order to elucidate how the marbling or IMF level influences the eating quality of pork.

After slaughtering, the cattle carcasses are often evaluated for their quality grades (QGs). In the world, countries such as USA, the beef carcass grading standard system has been developed from 1996 [13], and it has contributed to the facilitation of beef marketing by separating highly variable beef carcasses into groups with more uniform in quality and composition. For beef producers, the grade of a beef carcass is a very important factor because the economic value of their product basically relies on the grade. Likewise, at retail, the information on the grade of a primal or sub-primal cut is generally considered as an important factor for consumer’s buying decision [14].

In Korea, where the beef carcass grading system was estab lished in 1998 by National Livestock Cooperative Federation [15], the pork carcass grading system for evaluation of pork carcasses was firstly designed in the 2010 and then modified by Korean Institute for Animal Products Quality Evaluation in 2013 [16]. In the Korean pork carcass grading system, the QG of a pork carcass is evaluated based on some major criteria including; marbling level, meat and fat color, and texture of exposed *longissimus dorsi* (LD) muscle at the last rib (13th) and the 1st lumbar vertebrae. And three carcass grades such as; “grade 1+”, “grade 1”, and “grade 2” are usually formed. Among the aforementioned criteria, the marbling defined as the fat flecks (visible fat or IMF) within the muscle tissue, is considered as the most important criterion. Of which, the good, fine and poor marbling degrees correspond to the grade 1+, grade 1, and grade 2, respectively. The pork carcass grading system is therefore an essential tool to determine the differences in economic values of carcasses at the market place. While, consumers can use the information of carcass QG as a reference for selection and thus the pork producers must rely on the consumer’s preferences to produce highly acceptable pork.

For many meat markets such as Korea and Japan, the con sumers desire the high IMF pork [17]. To the best of our knowledge, however, no scientific information regarding the physicochemical composition, technological and eating qualities of pork as affected by the carcass QG is available. Thus, the present study aimed at evaluating the effect of carcass QG on the technological quality traits, fatty acids, flavor compounds and sensory characteristics of pork according to Korean carcass QG system. The findings of our study could be valuable information to give consumers an insight on how the meat quality differences relate to meat grades; and for meat producers in order to produce the pork that is highly accepted by consumers.

## MATERIALS AND METHODS

### Animals and samples preparation

A total of 51 crossbred ([Landrace×Yorkshire] ♀×Duroc ♂) (LYD) pigs (25 gilts and 26 barrows) with body weights of 100 to 120 kg at finishing ages of about 180 days collected from local farms in Korea were used in the present investigation. All the pigs were reared under same conditions and fed with a same commercial diet. The pigs were transported from the farms to a commercial slaughterhouse with a transporting time of about 1 to 2 h. After arriving, the pigs were laired in pens for 6 to 8 h with full access to water but fasted from food. Pigs were slaughtered following the commercial slaughtering process including electrical stunning, bleeding, scalding, dehairing, singeing, head and foots removing, eviscerating, washing and chilling. After chilling for 24 h in a chilling room (2°C±2°C), the carcasses were graded according to the Korean pork carcass grading system (KIAPQE, 2013) by an official meat grader. Based on the grading criteria (marbling, meat color, meat texture, fat color, and fat quality degrees) as described by Muhlisin et al [18], three different carcass QG groups including: grade 1+ (QG1+), grade 1 (QG1), and grade 2 (QG2), were formed. For each grade group, 10 carcasses were finally selected and transferred to a cutting room where the LD muscle from 1st to 13th thoracic vertebrae were collected from the left carcasses side and used for the meat quality analyses in the present study. The muscle samples were then trimmed off all visual fats and connective tissues, cut into sub-samples depending analyses. The pH, instrumental color, cooking loss, shear force, water holding capacity (WHC) and proximate composition were determined on the same sampling day (the day after slaughter) while, the samples used for fatty acids, flavor compounds and sensory evaluation were vacuum-packaged and stored at −20°C until use.

### pH measurement

The pH measurement was performed immediately after trimming by using a pH*K 21 (NWK-Technology GmbH, Kaufering, Germany) equipped with a stainless steel and solid-state probe. Before using, the pH meter was calibrated with pH 4.0 and 7.0 standards (NWK Technology., Ltd., Germany), and then the probe was inserted deeply into the muscle tissue. The pH values of each sample were the average of the three readings.

### Instrumental color measurement

The instrumental color traits were determined on 3 different locations of the freshly cut surface of each sample after 30 min blooming using a Minolta Chroma Meter CR-400 (Minolta Camera Co, Osaka, Japan). Prior to use, the device was standardized with a white plate (Y = 86.3, X = 0.3165, and y = 0.3242). Color was expressed according to the Commission International de l’Eclairage (CIE) system and reported as CIE L* (lightness), CIE a* (redness), CIE b* (yellowness), chroma and hue angle. In which the chroma and hue angle were calculated as (a*^2^+b*^2^)^0.5^ and tan^−1^ (b*/a*), respectively.

### Proximate composition

The proximate compositions (moisture, protein, and fat contents) were determined using a Food Scan Lab 78810 (Foss Tecator Co., Ltd., Hillerod, Denmark), as described in previous study [19]. Briefly, about 200 g of homogenized meat sample (each) was distributed onto the instrument’s round sample dish and loaded into the instrument’s sample chamber. Each sample was determined in triplicates.

### Cooking loss and Warner-Bratzler shear force value measurement

The cooking loss and Warner-Bratzler shear force (WBSF) were measured on the same steak (3.0-cm in thickness and weight of about 200 g) of each sample, as described in our previous work [20].

Following the cooking loss measurement, the cooked sam ples were used for the WBSF determination; for each sample, approximately 5 representative cores with an average diameter of 0.5 inches and length of at least 2 cm were removed parallel to the muscle fiber direction using a 0.5-inch metal corer. The WBSF values were obtained by completely cutting the cores in an Instron Universal Testing Machine (Model 4465, Instron Corp, High Wycombe, UK) using a crosshead speed of 200 mm/min and a 40 N load cell.

### Water holding capacity

The WHC of the muscle samples was measured following the procedure as described in our previous study [21] and by Han et al [22] with minor modifications. Briefly, after chopping and grinding using a mini grinder, approximately 0.51 g of each sample was taken and placed in a 2 mL ultra-centrifugal filter unit, inserted into an ultra-centrifugal filter device (Millipore Corp., Bedford, MA, USA), and then heated in an 80°C pre-heated water bath for 20 min. The heated samples were cooled at room temperature for 10 min and then centrifuged at 2,000×*g* for 10 min at 4°C. After centrifugation, the weight of ultra-centrifugal filter unit containing the cooked sample was recorded to determine the water loss. Each sample was analyzed in duplicates and the WHC percentage was calculated as a ratio of moisture to the water loss.

### Fatty acid profiles

Fats in samples were extracted using a solvent mixture of chloroform:methanol (2:1, v/v) as described by Folch et al [23] and then the extract was methylated using the procedure of Morrison and Smith [24]. The fatty acids were determined using a gas chromatography/flame ionization detector system (Model Star 3600, Varian Technologies, Palo Alto, CA, USA). The separation of fatty acids was performed on an Omegawax 205 fused silica bond capillary column (30 m×0.25 mm×0.25 μm film thickness; Supelco, Bellefonte, PA, USA) at a split ratio of 100:1. Nitrogen was the carrier gas in constant pressure mode at 16.0 psi and a flow rate of 1.0 mL/min. An aliquot of 2 μL of each sample was injected into the injection port, and the temperatures of injector and detector were set at 250°C and 260°C, respectively. The oven temperature was held at 50°C for 1 min, and then raised to 200°C at a rate of 25°C/min, and further increased to 230°C at a rate of 5°C per min. The fatty acids in samples were identified by comparing their retention times with those obtained from standard fatty acids. The results were expressed as relative percent (%) of total fatty acids based on total peak area.

### Volatile flavor compounds

The quality and quantity of volatile flavor compounds were determined using our previous method [25] with minor modifications. Briefly, the sample preparations (slicing manner and cooking conditions) were same as those used in the sensory evaluation as mentioned above. Immediately after cooking, 1 g of each sample was taken and placed into a 20-mL headspace vial and sealed with polytetrafluoroethylene-faced silicone septum for extraction. The extraction of volatile flavor compounds was done using solid-phase micro-extraction (SPME). A SPME device containing carboxen–polydimethylsiloxane (75 μm) fibre (Supelco, USA) was used and all the steps from extraction, absorption, desorption of the flavor compounds and fibre cleaning before/after each sample completion were done using a fully automated SPME sample preparation instrument (Model: AOC-5000 Plus) connected to gas chromatography (GC, Model: 7890B) with mass spectrophotometry (MS, Model: 5977B MSD, Agilent Technologies, Santa Clara, CA, USA). The SPME was carried out at 65°C and agitated at 250×rpm for 60 min. The GC/MS conditions set were same as those mentioned in the above cited literature [25]. The identifications of volatile compounds were performed by comparing their mass spectra with those already present in the Wiley registry of mass spectral data (Agilent Technologies, USA) and/or by comparing their retention times with those of authentic external standards. Approximated quantities of the volatile compounds were quantified by comparison of their peak areas with that of the 2-methyl-3-heptanone internal standard obtained from the total ion chromatogram using a response factor of 1.

### Sensory evaluation

The sample preparations and sensory evaluation were performed using the method of Seong et al [19] with minor modifications. Briefly, each muscle sample from each QG group was tested by six panels allocated in randomized block arrangement. The panels consisted of 12 members (four females and eight males) who were staff at the Animal Products Processing Division of National Institute of Animal Science, and they were chosen based on their previous experiences in sensory evaluation of meats. Each session had six panelists; each panelist evaluated six samples, and two sessions per day (10:30 am and 15:00 pm) were performed. Prior to use, the frozen vacuum-packed sub-samples were thawed in a cooling room (4°C) for 2 h. For each sample, 7 representative slices (50 mm×50 mm×4 mm) were prepared parallel to fiber direction, in which 1 slice was used for the sensorial color evaluation. For the sensorial color evaluation, the freshly-cut slices were evaluated after 30 min cutting (blooming), each the slice was passed through to all the panelists. The remaining 6 slices/sample were cooked on an open tin-coated grill for about 2 min and turned 30 s intervals. The cooking temperature was monitored using an infrared thermometer and was maintained at around 220°C. One set of grill was used where the grill was set to cook six slices of samples. Immediately after cooking, the samples were placed on individual plates and served to the panelists. Five major sensory traits such as; sensorial color of fresh meat, flavor, juiciness, tenderness and overall acceptability were used and each the sample was then evaluated for the aforementioned attributes using a 7-point scale (7 = extremely like; 6 = like very much; 5 = like moderately; 4 = neither like nor dislike; 3 = dislike moderately; 2 = dislike very much; and 1 = dislike extremely) as described by Meilgaard et al [26]. The panelists were asked to refresh their palate with drinking distilled water and salt-free crackers between samples. All sensory sessions were carried out in the sensory panel booth room equipped with white lighting.

### Statistical analysis

The obtained data were analyzed using Statistic Analysis System package (SAS Institute, Cary, NC, USA, 2007). Means and standard errors were calculated for the variables. The data were analyzed by using the General Linear Model procedure considering the QG as a main effect. Multiple mean comparisons were performed using Duncan’s multiple range test. The correlations between the chemical composition and quality traits with the QG were determined using Pearson’s linear correlation coefficient. The significance was defined at p<0.05.

## RESULTS AND DISCUSSION

### Effects of quality grade on meat quality characteristics

The effects of QG on the technological quality traits, instrumental color and proximate composition are presented in Table 1. The pH values of pork samples ranged from 5.67 to 5.71 with no significant differences among the three QG groups (p>0.05). In general, these ultimate pH values fell within the ranges (5.7 to 5.8) for the normal pork [27]. The cooking loss and WHC also were not significantly affected by the QG, but WHC increased as the QG increased. A significant difference in WBSF values occurred among the QG groups with lower values for the QG1+ (2.50 kgf) or QG1 (2.87 kgf) than for the QG2 (2.91 kg) (p<0.05). Yoo et al [28] reported lower pH values but higher cooking loss (34% to 35%) and WHC level (95% to 96%) for the LD muscle from the same pig breed at 24 h post-mortem compared with those of the current study.

The QG only affected the fat content, with significantly higher level for the samples of QG1+ (5.43%) than for the samples of QG2 (4.53%) (p<0.05) (Table 1). This finding agrees well with that of Brewer et al [5] and Cannata et al [29], who showed an increase in the fat content with increased marbling degree. When compared to our results, Jung et al [30] reported lower fat content (2.1% to 2.7%) for the LD muscle of pure breeds (Duroc, Landrace, and Large White) but Muhlisin et al [18] reported higher fat level (5.6% to 6.0%) for the LD muscle of Korean native black pigs. These contrasting results could be attributed to the different pig breeds used among the studies.

The visual appearance of pork such as color, is a key factor affecting the consumer purchasing decisions [7]. Our results showed that the QG considerably affected the color traits. For instance, the pork of QG1+ had higher L* (lightness), and lower a* (redness) values than the samples from QG 2 (p< 0.05). This was also confirmed by the results of Pearson’s linear correlation analysis (Table 2) in which the QG1+ was positively correlated to lightness (r = 0.861) whereas, the QG 2 was highly correlated to redness (r = 0.987). This could be related to the differences in the intramuscular fat content among the QG groups as mentioned above. In contrast to our results, Cannata et al [29] reported no effects of marbling degree on the instrumental color traits of pork LD muscle, which could be due to the fat content differences in samples between the two studies as they reported lower fat content (2.8% to 4.8%) than we did for the samples of LYD pigs.

### Effects of quality grade on fatty acid profiles

Table 3 shows the fatty acid composition expressed as % of total fatty acids. In general, the QG shows a negligible impact on the fatty acid composition as it did not result in differences in levels of the saturated (SFA) or unsaturated fatty acids (UFA), which agrees well with the findings of Cannata et al [29]. In the present study, the levels of most predominant fatty acids (e.g., C18:1n-9, C18:2n-6, and C18: 3n-3) detected were similar to those reported for the pork LD muscle by these authors but higher than those reported for the LD muscle of black pig breed [31]. Although the levels of individual fatty acids did not differ significantly among the QG groups, the total percent of UFA and polyunsaturated fatty acids (PUFA) were significantly different. Interestingly, the level of UFA was found significantly higher in the QG1+ (62.72%) compared to that (56.75%) of the QG 2 (p<0.05). Data similar to ours study for UFAs were reported by Cannata et al [29], who showed an increase in UFA content as the marbling level increased. The level of fatness has been found as an important factor affecting the fatty acid composition in meat tissues; a faster increase in the SFA and monounsaturated fatty acids (MUFA) fatty acids content with increased fatness [32]. However, our results showed no significant differences in SFA and MUFA contents among the carcass QGs (p>0.05). These contrasting observations could be attributed to the pig breed and genotype differences between the studies since these have been reported as a factor largely affecting the fatness and fatty acid composition in meat [32]. From the view of health benefit, the PUFA/SFA ratio for a healthy diet should be 0.40 or higher, while the ratio of n-6/n-3 fatty acids should be 4.0 or lower [33]. Our results showed that the n-6/n-3 ratios were significantly higher in the QG1+ than in the QG2, and they all were higher than recommended value of lower than 4.0. Furthermore, the PUFA/SFA ratio was higher in the QG1+ (0.69) than in the QG2 (0.32). Seong et al [31] reported a lower PUFA/SFA ratio (0.15) for the LD muscle of black pigs. Thus, it may be said that excepting the undesirable n-6/n-3 ratio, the pork of carcasses with higher QG presented the better fatty acid profiles than the ones with lower QGs.

### Effect of quality grade on flavor compounds

It is well recognized that the flavor is considered an important sensory trait for the eating quality of pork, and is primarily created by a variety of volatile flavor compounds in the Maillard reaction and thermally induced lipid oxidation during cooking process [34,35]. The effects of QG on the quality and quantity of volatile flavor compounds are presented in Table 4. A total of 47 flavor compounds including aldehydes (19), alcohols (4), ketones (3), hydrocarbons (7), pyrazines (5), sulfur-containing compounds (7), and furans (2), were detected from the samples of the three QG groups. The results of statistical analysis revealed that the QG significantly affected the amounts of eight of the 47 detected compounds (p<0.05). Regarding the aldehyde class, only two in the 19 compounds showed statistical differences among the QG groups. In particular, Hexanal and Benzaldehyde, the products produced from the oxidation process of C18:2n-6 and C18:3n-3, respectively [35], whose concentrations were significantly higher in the QG1+ than in the QG1 and QG2 (p<0.05).

It has been reported that the class of aldehydes associated with green, fatty and fruity odor notes at very low odor detection threshold (e.g., part per million) are important in the development of cooked pork’s flavor characteristics [36]. Our results showed that the total amount of aldehydes were significantly higher in the QG1+ (2.94 μg/g) than in the QG 1 (1.70 μg/g). This result could be related to the significantly higher UFAs content in the QG1+ compared to the other QG groups (Table 3). Furthermore, a positive correlation coefficient (r = 0.927) was also found between the aldehydes content and QG1+ (Table 2), meaning that increasing the QG results in an increase in aldehydes content in the cooked pork. Among the alcohols, only 1-octen-3-ol content showed significant differences with higher level for the QG1+ (0.13 μg/g) than for the QG1 (0.06 μg/g) and QG 2 (0.06 μg/g) (p<0.05). 1-Octen-3-ol is known to be formed from the oxidation of C18:2n-6 during cooking/heating, and it is associated with mushroom odor note [35]. Additionally, the total amount of alcohol classes was also significantly higher in the QG1+ (0.31 μg/g) than in the QG1 (0.11 μg/g) and QG2 (0.13 μg/g) (p<0.05).

Pyrazines are known to be formed from the Maillard re action between reducing sugars (e.g., ribose) and free amino acids [35], and possess the pleasant odor notes such as roasted and potato with low odor threshold [36]. Our results, however, showed no differences for all the pyrazines as well as total pyrazines content among the QG groups (p>0.05). This may suggest that the fatness or QG had no effects on the levels of flavor precursors such as free amino acids and carbohydrate contents which are important components for production of pyrazines in meat during cooking.

The sulfur-containing compounds such as thiophenes are known to be formed from the Maillard reaction, and are the important compounds in the cooked pork’s flavor development as they possess pleasant odor notes such as roasty, onion and meaty [36]. Only 3-thiophene acetic acid content was significantly higher in the QG2 than the QG1 (p<0.05). No statistical differences in the total sulfur-containing compounds content occurred among the three QG groups (p>0.05). This may again confirm that the QG seemed to have minor effects on the levels of flavor precursors such as free amino acids. Hydrocarbons, the products of the Maillard reaction and fatty acids oxidation, are high in odor detection threshold, thus contributing less significantly to flavor development of cooked meat [35]. Our results showed that only two compounds (1,3-dimethylbenzene and 2,5-octanedione), were significantly different among the QG groups (p<0.05). Similarly, only 2-dodecanone content was significantly affected by the QG (p<0.05). The ketones are usually formed from the oxidation of fatty acids, and they seem to trivially contribute to the cooked meat flavor because of their high odor-detection threshold.

In general, from the results obtained on the volatile flavor compounds, we observed that the QG seemed to show a greater effect on the concentrations of the UFAs-oxidized flavor compounds rather than on the compounds which are produced from the Maillard reaction between reducing sugars (e.g., ribose) and free amino acids. This is also consistent with those observed on the fatty acids results (Table 3).

### Effect of quality grade on eating quality

Consumer satisfaction is an important factor determining the quantity of meat that is purchased by consumers, and the most important aspect of meat quality is eating quality, usually defined as scores given by taste panelists. Table 5 shows that pork samples with different QGs had different sensory results (e.g., flavor, juiciness, tenderness, and acceptability scores). Interestingly, significantly higher flavor, juiciness and tenderness scores were given for the pork samples from the QG1+ than those for the samples from QG1 and QG2 by the panelists (p<0.05). The panelists also gave higher acceptability scores for the QG1+ followed by QG1 or QG2. Data similar to ours study for sensory quality were reported by Brewer et al [5] and Cannata et al [29] for pork. These authors reported that pork loin associated with higher marbling level were perceived by panelists to be juicier and tender than the leaner ones. The results indicating higher juiciness scores given for the QG1+1 could be related to its higher fat content (Table 1) which tends to increase the amount of perceived moisture in muscle thus improving juiciness of the pork (Cannata et al [29]). On the other hand, the significantly higher flavor scores given for the QG1+1 could be related to its higher amounts of flavor compounds such as aldehydes and alcohols etc. (Table 4). Furthermore, the Pearson’s correlation (Table 2) shows that the QG1+ was positively correlated to the flavor (r = 0.932), juiciness (r = 0.962), tenderness (r = 0.980), and overall acceptability (r = 1.000). This means that increasing the carcass QG improves the eating quality of pork.

## CONCLUSION

The present study, for the first time investigated the relationships between the technological quality traits, fatty acid profiles, flavor components and eating quality of pork according to Korean carcass QG system. For the meat quality traits, the pork samples of QG1+ presented significant lower shear force, lightness and yellowness values compared to the samples of the QG1 and QG2. Although, the QG did not affect the levels of individual fatty acids, however, it did influence the total UFAs content. Except the undesirable n-6/n-3 ratio, the pork of QG1+ presented the better fatty acid profiles than the ones from the QG1 or QG2. A total of 47 volatile flavor compounds were detected from the cooked pork samples, and amongst eight compounds showed statistical differences among the three grade groups. We observed that the QG showed a greater effect on the amounts of UFAs-derived flavor compounds rather than did on the Maillard reaction-derived compounds (e.g., pyrazines and sulfur-containing compounds). The QG1+ was positively correlated to the flavor, juiciness, tenderness and acceptability scores. Based on the results obtained from our investigation, it may be concluded that pork with different QGs had different quality characteristics, fatty acids composition and quantities of flavor compounds as well as eating quality.

## Figures and Tables

**Table 1:** Technological quality, color traits and chemical compositions of pork meat as affected by quality grade

Items	QG1+	QG1	QG2
Technological quality traits			
pH	5.71±0.02	5.67±0.02	5.68±0.02
Cooking loss (%)	28.79±0.44	30.96±1.06	31.35±0.86
Water holding capacity (%)	64.37±0.91	63.08±0.63	62.83±0.91
Shear force (kgf)	2.50±0.06b	2.87±0.07^b^	2.91±0.08^a^
Chemical compositions			
Moisture (%)	70.66±0.34	70.57±0.41	70.81±0.29
Fat (%)	5.43±0.35^a^	5.14±0.36^ab^	4.53±0.26^b^
Protein (%)	24.73±0.61	24.69±0.66	24.92±0.60
Collagen (%)	2.06±0.31	2.24±0.34	2.02±0.30
Instrumental colors			
CIE L*	57.42±0.46^a^	56.48±0.56^a^	54.66±0.74^b^
CIE a*	5.39±0.16^b^	5.56±0.16^b^	6.39±0.21^a^
CIE b*	4.68±0.29^a^	3.95±0.18^b^	4.57±0.21^ab^
Chroma	8.29±0.62^a^	6.70±0.21^b^	7.65±0.26^ab^
Hue angle	35.56±0.97	35.78±1.05	36.86±1.22

QG1+, quality grade 1+; QG1, quality grade 1; QG2, quality grade 2.

a,b Means within a same row with different letters differ significantly at (p<0.05).

**Table 2:** Correlation coefficients between the quality traits with quality grades

Items	QG1	QG1+	QG2
Total aldehydes	−0.789	0.927*	−0.138
Total alcohols	−0.655	0.982*	−0.327
Total pyrazines	0.564	0.434	0.397
Total sulfur-containing compounds	0.690	0.086	0.280
Total hydrocarbons	0.729	0.899*	−0.069
Total ketones	0.791	0.693	0.277
Total furans	0.500	−0.500	0.500
C14:0	0.970	−0.694	−0.276
C16:0	0.567	0.799	0.430
C16:1n7	−0.263	0.967	−0.704
C18:0	0.617	−0.990*	0.373
C18:1n9	−0.474	−0.525	0.230
C18:1n7	0.783	−0.930*	0.147
C18:2n6	−0.074	0.490	−0.782
C18:3n6	−0.229	0.957*	−0.729
C18:3n3	0.883	−0.034	−0.848
C20:1n9	−0.319	−0.661	0.980
C20:4n6	−0.176	0.940*	−0.765
C20:5n3	−0.133	0.925*	−0.792
C22:4n6	−0.065	0.897	−0.832
C22:6n3	−0.238	0.960*	−0.722
SFA	0.607	−0.992*	0.385
UFA	−0.607	0.992*	−0.385
MUFA	−0.218	−0.736	0.954
PUFA	−0.097	0.911*	−0.813
n3	0.204	0.746	−0.950
n6	−0.102	0.612	−0.811
n6/n3	−0.250	0.963*	−0.714
MUFA/SFA	−0.790	−0.136	0.926
PUFA/SFA	−0.224	0.956*	−0.732
pH	−0.693	0.971	−0.277
CIE L*	0.181	0.861*	−0.942
CIE a*	−0.356	−0.631	0.987*
CIE b*	−0.990	0.616	0.374
Chroma*	−0.917	0.805	0.112
Hue angle	−0.357	−0.631	0.987
Moisture	−0.786	−0.143	0.929
Fat	0.201	0. 874*	−0.949*
Protein	−0.634	−0.352	0.987
Collagen	0. 598	−0.345	−0.640
Water holding capacity	−0.363	0.988	−0.625
Cooking loss	0.373	−0.990	0.617
Shear force	0.421	−0.996*	0.575
Color	0.998	−0.551	−0.447
Flavor	−0.151	0.932*	−0.870*
Juiciness	−0.246	0.962*	−0.861*
Tenderness	−0.320	0.980*	−0.862*
Acceptability	−0.485	1.000*	−0.815*

QG1+, quality grade 1+; QG1, quality grade 1; QG2, quality grade 2.

* Significant at p<0.05.

**Table 3:** Relative percentage (%) of fatty acids in pork *longissimus dorsi* muscle as affected by quality grade

Items	QG1+	QG1	QG2
C14:0	1.32±0.64	1.55±0.12	1.72±0.21
C16:0	24.69±4.37	25.95±0.69	27.74±1.97
C16:1n7	2.97±0.28	2.98±0.26	1.97±0.48
C18:0	11.27±2.35	11.23±0.33	13.79±2.03
C18:1n9	38.67±0.45	35.66±3.79	39.56±0.96
C18:1n7	0.17±0.10	0.19±0.03	0.24±0.04
C18:2n6	19.03±8.99	17.36±2.99	12.05±0.99
C18:3n6	0.16±0.11	0.12±0.03	0.08±0.00
C18:3n3	0.39±0.07	0.39±0.04	0.45±0.06
C20:1n9	0.64±0.27	0.59±0.18	0.84±0.23
C20:4n6	4.10±2.93	3.42±1.19	1.28±0.21
C20:5n3	0.08±0.05	0.09±0.04	0.04±0.00
C22:4n6	0.44±0.30	0.39±0.26	0.22±0.02
C22:6n3	0.05±0.04	0.07±0.00	0.03±0.00
SFA	37.28±7.36	38.74±1.14	43.25±4.08
UFA	62.72±7.36^a^	61.26±1.14^ab^	56.75±4.06^b^
MUFA	38.46±5.11	39.42±3.33	42.61±4.79
PUFA	24.26±12.48^a^	21.83±4.46^ab^	14.14±1.25^b^
n3	0.53±0.15	0.55±0.00	0.52±0.06
n6	23.74±12.32	21.28±4.47	13.62±1.18
n6/n3	44.79±11.34^a^	38.60±8.28^ab^	26.19±1.75^b^
MUFA/SFA	1.29±0.55	1.01±0.06	0.98±0.18
PUFA/SFA	0.65±0.01^b^	0.56±0.01^ab^	0.32±0.03^a^

QG1+, quality grade 1+; QG1, quality grade 1; QG2, quality grade 2; SFA, saturated fatty acids; UFA, unsaturated fatty acids; MUFA, mono unsaturated fatty acids; PUFA, poly unsaturated fatty acids.

a,b Means within a same row with different letters differ significantly at (p<0.05).

**Table 4:** Volatile aroma compounds (μg/g) of cooked pork longissimus dorsi muscle as affected by quality grade

Items	Retention time (min)	QG1+	QG1	QG2	Identification method1)
Aldehyde					
Propanal	1.701	ND	0.05±0.00	0.07±0.01	MS+STD
Butanal	2.140	ND	0.02±0.01	0.03±0.02	MS+STD
2-methyl-heptanal	3.279	0.05±0.00	0.04±0.01	0.04±0.02	MS+STD
Hexanal	6.075	1.49±0.17^a^	0.43±0.06^b^	0.47±0.07^ab^	MS+STD
Heptanal	9.244	0.12±0.02	0.12±0.03	0.14±0.06	MS+STD
E-2-heptenal	10.737	ND	0.02±0.00	0.02±0.01	MS+STD
Benzaldehyde	10.851	0.61±0.01^a^	0.43±0.03^b^	0.45±0.05^b^	MS+STD
Octanal	11.904	0.22±0.03	0.18±0.05	0.23±0.08	MS+STD
E-2-octenal	13.168	0.03±0.00	0.04±0.01	0.04±0.01	MS+STD
E,E,2,4-decadienal	13.869	ND	0.01±0.00	0.03±0.01	MS+STD
Nonanal	14.179	0.68±0.11	0.43±0.09	0.51±0.17	MS+STD
E-2-nonenal	15.513	ND	0.02±0.01	0.02±0.01	MS+STD
Decanal	16.207	0.03±0.00	0.03±0.01	0.07±0.03	MS+STD
E-2-decenal	17.253	ND	0.02±0.01	0.08±0.04	MS+STD
Undecanal	18.065	ND	0.02±0.00	0.08±0.05	MS
2-undecenal	19.051	0.02±0.01	0.04±0.01	0.07±0.03	MS
Tridecanal	21.429	0.03±0.01	0.03±0.01	0.07±0.03	MS
Pentadecanal	22.966	ND	0.05±0.01	0.03±0.00	MS
Tetradecanal	25.836	ND	0.33±0.05	0.23±0.09	MS
Total aldehydes		2.94±0.21^a^	1.70±0.23^b^	2.17±0.34^ab^	
Alcohols					
1-pentanol	5.008	0.03±0.00	0.02±0.00	0.02±0.01	MS+STD
1-heptanol	11.079	0.03±0.01	0.05±0.01	0.05±0.02	MS+STD
1-octen-3-ol	11.326	0.13±0.01^a^	0.06±0.01^b^	0.06±0.01^b^	MS+STD
Benzyl alcohol	12.604	0.03±0.01	0.06±0.00	0.08±0.02	MS
Total alcohols		0.21±0.02^a^	0.11±0.02^b^	0.13±0.04^b^	
Pyrazines					
Methylpyrazine	6.890	0.06±0.01	0.05±0.01	0.04±0.00	MS+STD
2,5-dimethyl pyrazine	9.505	0.18±0.03	0.20±0.01	0.12±0.03	MS+STD
2,3-dimethylpyrazine	9.648	0.03±0.01	0.04±0.00	0.04±0.00	MS+STD
Trimethyl pyrazine	11.821	0.13±0.02	0.13±0.01	0.07±0.02	MS
3-methyl-2,5-dimethyl pyrazine	13.540	0.07±0.01	0.06±0.00	0.06±0.00	MS
Total pyrazines		0.45±0.07	0.47±0.02	0.23±0.09	
Sulfur-containing compounds					
Carbon disulfide	1.864	ND	0.00±0.00	0.06±0.01	MS
3-thiophene-carboxaldehyde	11.645	0.05±0.01	0.04±0.00	0.05±0.01	MS
2-thiophene methanol	12.512	ND	0.06±0.00	0.06±0.01	MS
Dimethyl tetrasulfide	16.495	0.03±0.02	0.03±0.01	0.02±0.00	MS
3-thiophene acetic acid	17.139	0.02±0.01^ab^	0.01±0.00^b^	0.05±0.02^a^	MS+STD
3-phenyl thiophene	19.822	0.87±0.11	0.41±0.18	1.22±0.55	MS+STD
2-phenyl thiophene	19.988	0.55±0.07	0.43±0.14	0.73±0.31	MS+STD
Total sulfur-containing compounds	1.51±0.20	0.74±0.30	2.08±0.86		
Hydrocarbons					
Ethylbenzene	7.966	0.03±0.00	0.02±0.00	0.02±0.00	MS
1,3-dimethylbenzene	8.245	0.13±0.01^a^	0.05±0.02^b^	0.08±0.02^ab^	MS
2,5-octanedione	11.461	0.13±0.02^a^	0.03±0.01^b^	0.04±0.01^b^	MS
3-methyl-butanamide	12.037	0.02±0.00	0.01±0.00	0.02±0.00	MS
Dodecane	16.081	0.05±0.01	0.03±0.01	0.04±0.01	MS
Tridecane	17.917	0.04±0.02	0.02±0.01	0.06±0.01	MS
Tetradecane	19.636	0.04±0.01^a^	0.02±0.00^b^	0.04±0.01^a^	MS
Total hydrocarbons		0.40±0.03^a^	0.15±0.04^b^	0.26±0.05^b^	
Ketones					
Acetophenone	13.344	0.03±0.01	0.03±0.01	0.03±0.01	MS
2-decanone	15.916	0.05±0.00^b^	0.02±0.01^b^	0.07±0.02^a^	MS+STD
2-tridecanone	21.168	ND	0.02±0.01	0.02±0.01	MS
Total ketones		0.09±0.01	0.05 0.01	0.08±0.03	
Furans					
2-pentyl furan	11.569	0.05±0.01	0.12±0.09	0.09±0.05	MS+STD
2-n-octyl furan	17.797	ND	0.09±0.04	0.05±0.01	MS+STD
Total furans		0.05±0.01	0.13±0.06	0.13±0.05	

QG1+, quality grade 1+; QG1, quality grade 1; QG2, quality grade 2; ND, not detected.

1) Identification method: The compounds were identified by either mass spectra (MS) from library or authentic standards (STD).

a,b Means within a same row with different letters differ significantly at (p<0.05).

**Table 5:** Mean scores (7-points scale) for sensorial traits of pork meat as affect by quality grade

Items	QG1+	QG1	QG2
Color	4.47±0.10	4.62±0.09	4.48±0.09
Flavor	4.33±0.07^a^	4.02±0.08^b^	3.84±0.06^b^
Juiciness	3.51±0.07^a^	3.33±0.08^ab^	3.26±0.07^b^
Tenderness	4.05±0.10^a^	3.44±0.09^b^	3.28±0.08^b^
Acceptability	3.97±0.06^a^	3.47±0.08^b^	3.46±0.07^b^

QG1+, quality grade 1+; QG1, quality grade 1; QG2, quality grade 2.

Means±standard errors; the mean values were calculated using 7-point scale (7 = extremely like; 6 = like very much; 5 = like moderately; 4 = neither like nor dislike; 3 = dislike moderately; 2 = dislike very much; and 1 = dislike extremely).

a,b Means within a same row with different letters are significantly different (p<0.05).
